# Making cups and rings: the ‘stalled-wave’ model for macropinocytosis

**DOI:** 10.1042/BST20231426

**Published:** 2024-08-28

**Authors:** Robert R. Kay, Judith E. Lutton, Jason S. King, Till Bretschneider

**Affiliations:** 1 MRC Laboratory of Molecular Biology, Cambridge CB2 0QH, U.K; 2 Department of Computer Science, University of Warwick, Coventry CV4 7AL, U.K; 3 Department of Biomedical Sciences, Western Bank, Sheffield S10 2TN, U.K.

## Abstract

Macropinocytosis is a broadly conserved endocytic process discovered nearly 100 years ago, yet still poorly understood. It is prominent in cancer cell feeding, immune surveillance, uptake of RNA vaccines and as an invasion route for pathogens. Macropinocytic cells extend large cups or flaps from their plasma membrane to engulf droplets of medium and trap them in micron-sized vesicles. Here they are digested and the products absorbed. A major problem — discussed here — is to understand how cups are shaped and closed. Recently, lattice light-sheet microscopy has given a detailed description of this process in *Dictyostelium* amoebae, leading to the ‘stalled-wave’ model for cup formation and closure. This is based on membrane domains of PIP3 and active Ras and Rac that occupy the inner face of macropinocytic cups and are readily visible with suitable reporters. These domains attract activators of dendritic actin polymerization to their periphery, creating a ring of protrusive F-actin around themselves, thus shaping the walls of the cup. As domains grow, they drive a wave of actin polymerization across the plasma membrane that expands the cup. When domains stall, continued actin polymerization under the membrane, combined with increasing membrane tension in the cup, drives closure at lip or base. Modelling supports the feasibility of this scheme. No specialist coat proteins or contractile activities are required to shape and close cups: rings of actin polymerization formed around PIP3 domains that expand and stall seem sufficient. This scheme may be widely applicable and begs many biochemical questions.

## Introduction

The plasma membrane is a permeability barrier that retains macromolecules and metabolites within cells but equally blocks nutrients from entering. These must be imported either by specialist transporters or by endocytosis [1]. Clathrin-mediated endocytosis is well-understood: it is based on the self-assembly of clathrin coats which — with the cooperation of many other proteins — pinch off tiny vesicles from the plasma membrane [2]. It efficiently takes up membrane proteins and ligands bound to specific receptors, plus small amounts of fluid.

The major form of fluid endocytosis in many cells — macropinocytosis — is quite different [3–8]. It produces fewer, but much larger vesicles, which can be 5 μm in diameter and have a volume more than 10 000 times that of a coated vesicle, or the tiny vesicles produced in other micro-endocytic pathways [9]. It is non-selective, allowing cells to take up large quantities of medium, including proteins, cell fragments and viruses. After uptake, medium passages through the endo-lysosomal system and its contents are digested and absorbed. Active cells, such as dendritic cells, macrophages or *Dictyostelium* amoebae, produce just a handful of macropinosomes per minute, yet can take up their own volume in a couple of hours [10–13].

Macropinocytosis spans the metazoa from coral to mammals as well as being found in amoebae [6,14]. It probably evolved for feeding, as seen today in amoebae and certain cells in mammals, such as those of the pre-implantation embryo and placenta [15,16]. More ominously cancer cells can feed on serum proteins by macropinocytosis, giving them a growth advantage over normal cells [17,18]. Macropinocytosis is also crucial for immune surveillance, RNA vaccine and pathogen entry into cells and may allow the spread of protein aggregates in neurodegeneration [8]. Macropinocytosis is generally stimulated by growth factors and macropinocytic cups are thought to reciprocally amplify growth-factor signal transduction [19].

**Figure 1:**
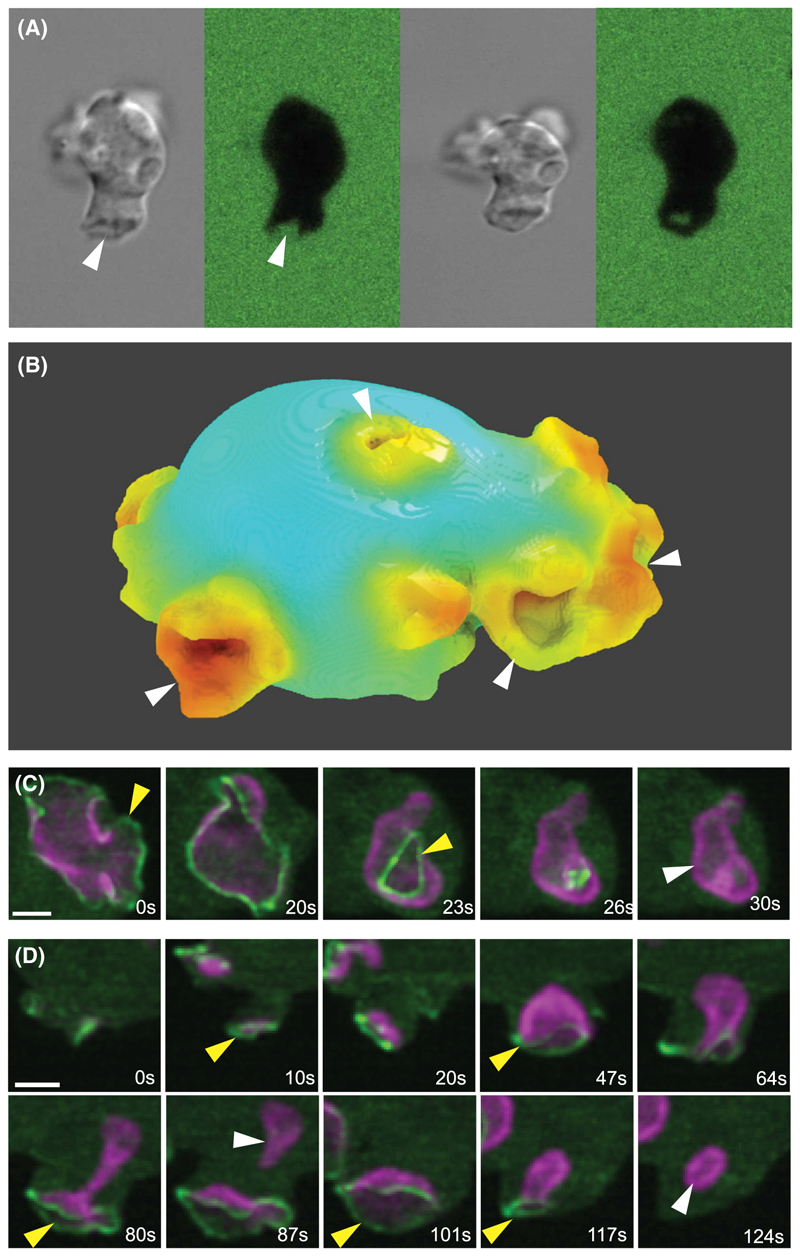
Macropinocytosis in *Dictyostelium* amoebae. **(A)** Engulfment of fluorescent medium by an amoeba into a macropinosome. An amoeba bathed in fluorescent FITC-dextran extends a macropinocytic cup (arrowed), which closes to yield a macropinosome. Viewed by confocal microscopy in DIC and fluorescence. Image courtesy of Douwe Veltman. **(B)** A rendered view of a *Dictyostelium* cell with several macropinocytic cups (arrowed) obtained from lattice light-sheet microscopy. The cell is expressing reporters for F-actin and PIP3, with F-actin intensity mapped. Image courtesy of Daniel Moore. (**C** and **D**) A ring of Scar/WAVE (yellow arrows) is recruited at the lip of cups during their expansion and subsequent closure at lip or base. (**C**) shows an en face view of a large cup closing at the lip (0–26 sec) to release a macropinosome (white arrow; 30 sec) still retaining its PIP3. (**D**) shows a side view of a cup first expanding (0–47 sec), then deepening and closing at the base to release a macropinosome (87 sec). Note that the Scar/WAVE remains at the lip during closure, not at the site of constriction (80 sec). The PIP3 and Scar/WAVE remaining at the plasma membrane then quickly form another microsome (124 sec) whose closure extinguishes the PIP3 domain. Scar/WAVE reporter = green; PIP3 reporter = purple. Scale bar = 2 μm. Lattice light sheet microscopy, from [34].

Macropinocytosis is still poorly understood, despite its discovery nearly 100 years ago [3]. Macropinosomes in mammalian cells form by the closure of cups or flaps of the plasma membrane that are extended by actin polymerization [4,20,21], while in *Dictyostelium* amoebae the canonical macropinocytic structure is a cup (Figure 1) [13]. How cups are shaped and closed is the focus of this review. There is no particulate guide as in phagocytosis, nor are coat proteins analogous to clathrin known. However, PI3-kinase and its product, the signalling phosphoinositide PIP3, is crucial in immune cells [22–24] and amoebae [25,26], where it forms an intense domain at the heart of cups [27,28].

**Figure 2:**
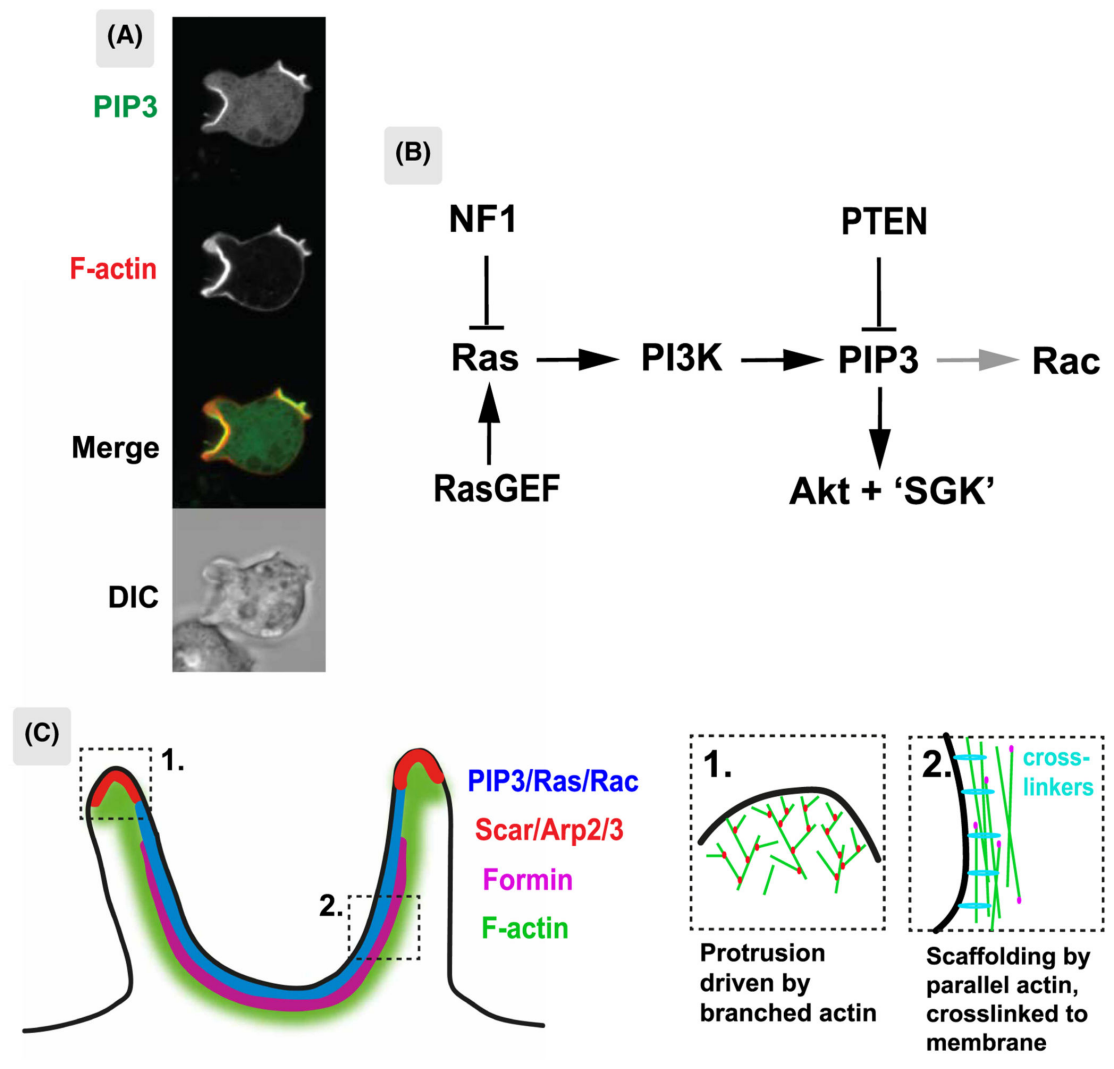
Macropinocytic cup organization. Cups are organized around a domain of PIP3, active Ras and active Rac in the plasma membrane and are linked to a specialized F-actin scaffold. (**A**) PIP3 domains in the plasma membrane of a *Dictyostelium* cell. PIP3 domains are readily visible in growing amoebae with each representing a macropinocytic cup. Cells are around 10 μm in diameter and domains can be several microns across. The cell is expressing reporters for PIP3 (PH-domain of PkgE-GFP) and F-actin (mCherry-LimE). Confocal microscopy, from [33]. (**B**) Outline of the conserved Ras/PIP3/Akt module of growth factor signalling that underpins PIP3 domains and macropinocytosis in *Dictyostelium*. This circuit is spontaneously active in PIP3 domains — no external signal is known to be required — and is genetically essential for fluid uptake by macropinocytosis (Table 1). Only selected components are shown; others include further RasGAPs, Rac, PKD1 and TORC2 that activate Akt and ‘SGK’ and the many downstream effectors, such as proteins binding to PIP3 or phosphorylated by Akt. NF1, Neurofibromatosis-1; RasGEF, Ras activating protein; the relevant one(s) in *Dictyostelium* are not known; PI3K, PI3-kinase of which there are five Ras-activated ones in *Dictyostelium*; PIP3 is an ether-linked (plasmanyl) lipid in *Dictyostelium* [62]. PTEN is a phosphatase reverting PIP3 to PI4,5P2; Akt and SGK are PIP3-activated protein kinases, with the *Dictyostelium* SGK homologue (PKBR1) being myristoylated and so persistently recruited to the plasma membrane [63]. *Dictyostelium* has multiple Rac proteins, with those relevant to macropinocytosis not fully known. (**C**) Schematic view of macropinocytic cups in *Dictyostelium* cells. Cups are marked by a PIP3 domain with its boundary just short of the lip and supported from top to bottom by a continuous F-actin scaffold, to which the membrane is attached by proteins such as myosin-1 and talin. Scar/WAVE and Arp2/3 are recruited around the domain boundary and catalyze dendritic actin polymerization, which pushes outwards on the membrane, while formins, which catalyze linear F-actin are found towards the base. The insets show detail of the actin cytoskeleton at the lip and base of the cup. Largely based on lattice light sheet microscopy and cryo-electron tomography [34,38].

Macropinocytosis is facultative in *Dictyostelium* cells — they prefer bacteria, but if a nutritious medium is provided instead, they switch to macropinocytosis via a defined genetic program [29,30]. There is good evidence that the classic Ras/PIP3/Akt signalling pathway, which is spontaneously active in macropinocytic *Dictyostelium* cells, is essential to create PIP3 domains capable of efficient macropinocytosis (Table 1, Figure 2). For instance, deletion of all five Ras-activated PI3-kinases abolishes macropinocytosis, whereas activation of Ras by deletion of the RasGAP NF1 (which inactivates Ras) increases the extent of PIP3 domains and macropinocytosis several fold [26,31]. With this genetic foundation, attention has focussed on how PIP3 domains function in macropinocytosis.

## Light sheet microscopy: observations and inferences

Macropinocytic cups, although large, are actually difficult to image with fluorescent reporters in their fullness and over their lifetime. A detailed description only became possible recently thanks to lattice light sheet microscopy [32], which allows full cell volumes to be acquired every few seconds at low light doses and over 10-min periods [20,21,33,34]. Using reporters for PIP3 and F-actin, macropinocytic cups in *Dictyostelium* cells can be seen growing from small origins, deepening, moving around on the cell surface, splitting, fusing, fading away and in general being morphologically ill-disciplined. Yet they are clearly effective, since most close to release one or several macropinosomes. These retain an F-actin coat and the PIP3 reporter for some seconds, before both disappear as the vesicle progresses through the endocytic pathway.

### Insights from the structure of cups

Several inferences can be drawn from mapping phosphoinositide lipids and cytoskeletal and signalling proteins in macropinocytic cups [34].

First, cups are defined by PIP3 domains (Figures 1 and 2), which mark them throughout their life and occupy their inner surface roughly up to the lip. The PIP3 signal is graded — highest at the middle/bottom of the cup and dropping towards the edge. PI3,4P2, which is produced by dephosphorylation of PIP3 and is an important signal in its own right, is barely detectable in PIP3 domains, though levels rise rapidly in macropinosomes after closure [28]. Conversely there is a reverse gradient of PI4,5P2, the precursor of PIP3, which is higher outside cups than within them. Similarly, PTEN, which reverts PIP3 to PI4,5P2 is excluded from cups but found on the surrounding membrane [35]. Together with the localization of PI3-kinase to PIP3 domains [26], these observations imply that macropinocytic cups are sites of intense, localized PIP3 production, and that PIP3 diffuses out from them to the rest of the plasma membrane, where it is reverted to PI4,5P2 by PTEN.

Second, PIP3 domains create a ring of actin polymerization around themselves that can be visualized using a reporter for the Scar/WAVE complex, which activates Arp2/3 and hence dendritic actin polymerization [33,34] (Figure 1C). The Arp2/3 complex [34,36] and VASP [37] are also recruited to this ring, while Leep1, a potential inhibitor of Scar/WAVE, is directed to the interior of cups by binding PIP3 [36]. The resulting ring of dendritic actin polymerization drives a hollow ring of membrane outwards, to form a cup.

Cryo-electron tomography confirms that the cytoskeleton in the outer part of PIP3 domains consists largely of dendritic F-actin with growing ends directed against the membrane and so applying outwards force, while the centre has more linear actin filaments parallel to the membrane [38]. These reinforce the cup without applying outward force and are catalyzed by formins [39–41].

Third, this spatially-organised actin polymerization creates an F-actin scaffold that envelopes cups from top to bottom, shaping and supporting them at all stages of their evolution [34]. We propose that this scaffold replaces the need for specialist coat proteins analogous to clathrin.

Fourth, often when cups close the membrane can be seen to abruptly detach and jump away from the F-actin scaffold (delaminate). This implies that the membrane is under tension, which must be imposed by the scaffold and released when the membrane delaminates [34]. In areas of negative curvature — the inner face of cups — this tension will tend to detach the membrane from the F-actin scaffold. Since this does not normally happen until the cup closes, we infer that cross-linking proteins must hold the two together. Candidates include myosin-1 proteins, which are greatly enriched in cups, particularly those that bind PIP3 and the FERM-domain protein talin, which also localizes to cups [42,43].

These observations paint a physical picture of a macropinocytic cup: a flexible, elastic membrane tightly linked to an F-actin scaffold which applies outward force along a ring at the lip of the cup, stretching the membrane and eliciting a force from membrane tension in return.

### How cups expand and close: the stalled wave model

Individual cups can be followed by microscopy from origin through to closure and the shape and area of their PIP3 domains measured (Figure 1C). Cups arising *de novo* first appear as small PIP3 concentrations at a site of actin polymerization. As the PIP3 domain expands it drives a wave of actin polymerization across the plasma membrane, capturing membrane and so enlarging the cup. Thus, cups grow because PIP3 domains expand.

Eventually the PIP3 domain slows and stops expanding — the surface area of the domain does not increase, or it even decreases — and the actin wave slows and stalls. The cup now closes. A key proposition of the model is that cup closure is caused by the actin wave stalling.

After the wave stalls, actin polymerization continues at the rim of the cup but now under the same ring of membrane. It elongates the cup, increasing tension within its membrane. This tension is not easily relieved by dragging membrane into the cup, due to the friction between membrane and scaffold; and we assume it is not relieved by exocytosis of membrane into the cup, despite the rapid cycling of the plasma membrane in *Dictyostelium* cells and their ability to rapidly change their surface area [44,45]. To preserve its surface area, the cup must therefore narrow. The force of actin polymerization extending the cup is directed outwards, but the inward reaction force can be transmitted to the membrane within the cup by retrograde actin flow, pulling it into the cell. Lengthening, narrowing and deeper penetration of the cup into the cell are all observed.

Macropinocytic cups can close in two ways. Some close at the lip: the lip turns inwards, possibly bent by membrane tension or due to slippage of the domain boundary into the cup, and the orifice then closes like a sphincter. Most of the PIP3 domain is included in the macropinosome and macropinocytosis is extinguished. Since macropinocytosis is resistant to inhibition of myosin-II, lip closure appears not to be driven by a myosin-II purse-string, as proposed for phagocytic cups [46]. The Scar/WAVE complex remains at the lip of the cup throughout, arguing that closure is driven by continued actin polymerization (Figure 1C).

Otherwise cups close at the base: the cup deepens and narrows and eventually constricts and seals off a macropinosome. Some PIP3 remains in the plasma membrane and can spawn further macropinosomes. Scar/WAVE remains at the lip of the cup, but not at the site of closure, implying that closure is driven remotely by actin polymerization at the lip. This stretches the membrane within the cup, increasing membrane tension, with the inward component of this causing constriction of the cup and eventual delamination of the membrane, sealing off a macropinosome.

### A basic model

The observations and inferences outlined above led to a simple model for how macropinocytic cups form and close [34] based on the following proposals:

- A ring of dendritic actin polymerization is formed around PIP3 domains producing outward force and a cup-shaped F-actin scaffold.
- Cups are shaped by this F-actin scaffold.
- The membrane of cups is strongly cross-linked to the scaffold allowing local increases of tension due to actin polymerization.
- Closure of cups is caused by the PIP3 domain/actin wave stalling while actin polymerization continues at the lip.

**Figure 3:**
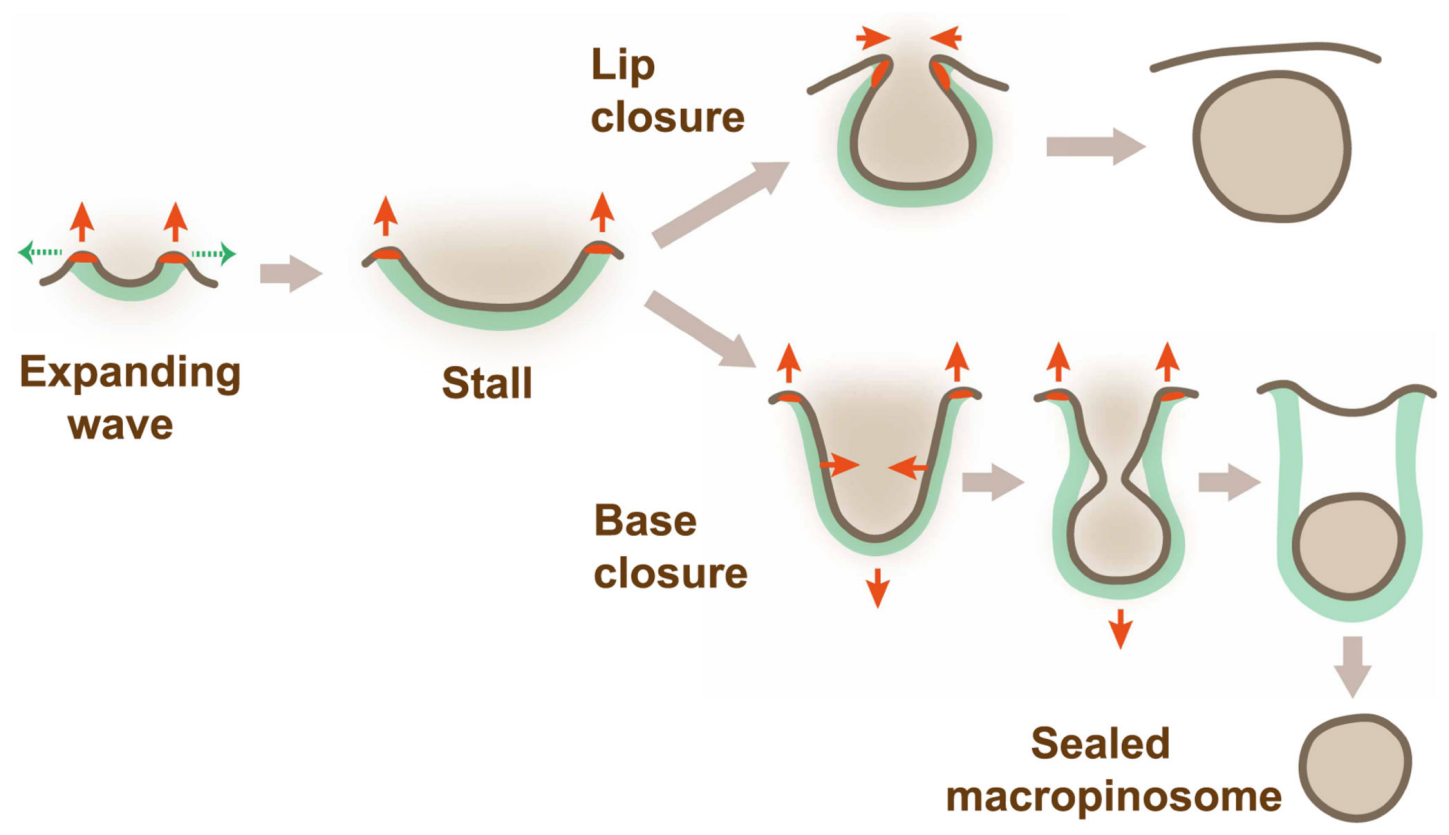
A conceptual model for macropinocytic cup formation and closure. ‘The model is based on PIP3 domains that activate a ring of actin polymerization around themselves. The force of this actin polymerization under the membrane, together with the tension it creates in the membrane, shape the cup as it grows and close it when it stalls.’ Initially (‘Expanding wave’) a macropinocytic cup starts as a small domain of PIP3 with a ring of actin polymerization around it, which exerts outward force normal to the membrane. The reaction force is applied to the F-actin scaffold, tending to drive it into the cell. As the PIP3 domain expands, so the wave of actin polymerization travels along the membrane capturing it into the cup. When the domain stalls, actin polymerization remains under the same area of membrane, deepening the cup and stretching the membrane within it. In lip closure the lip turns inwards and is constricted by continued actin polymerization. In base closure, the F-actin scaffold transmits downward force to the membrane by retrograde flow of F-actin, and the inward component of the increasing membrane tension constricts the cup and eventually leads to delamination of the membrane and closure of a macropinosome. This mechanically-based model reproduces many of the behaviours observed by microscopy, including lip and base closure [34]. Red arrows show forces; red patches in the membrane show the zone of dendritic actin polymerization; green dashed arrows show direction of expansion of the PIP3 domain; green fill = F-actin scaffold; dark brown = plasma membrane.

With suitable parameters, this model can reproduce macropinocytic cups expanding and then closing at lip or base when they stall (Figure 3). The earlier model of Saito and Sawai [47] is also based on a ring of actin polymerization which expands and then stalls. It differs in not allowing tension gradients to build up in the cup and treating the PIP3 domain as a reaction-diffusion system, giving a much richer set of behaviours. Despite these differences, this model also reproduces the essential features observed microscopically, showing that these are a robust consequence of the initial assumptions.

## Discussion

Macropinocytic cups are self-organizing structures formed on the scale of microns by the actin cytoskeleton and the plasma membrane. The stalled wave model provides an experimentally-supported scheme for how they are formed in amoebae, based on domains of PIP3 that attract a ring of actin polymerization around themselves and close when they stop expanding [34]. It is not yet known whether this model, which requires much further testing, extends to other cell types and other cupped structures. This is discussed below.

Macropinocytosis in mammalian cells, as in *Dictyostelium*, depends on PIP3, which likewise forms intense domains in the plasma membrane where they form [48]. Whether these domains also attract dendritic actin polymerization and Scar/WAVE to their edge is unknown. Unlike *Dictyostelium*, macropinosomes in mammalian cells can also be produced by flaps of membrane that fold back on themselves [21], which is not readily explained by the stalled wave model.

Phagocytic cups can ingest particles in a ‘zippering’ process of successive receptor engagements across the particle’s surface [49]. However, since ‘spacious phagosomes’ form without a particle as template, it appears that phagocytic cups are also capable of spatial self-organization [50]. Phagocytic cups in both mammalian cells and *Dictyostelium* have a core PIP3 domain [28,51], with actin presumed to polymerize at its periphery. Finally, circular dorsal ruffles are very large, shallow cups formed on the dorsal surface of cells in response to growth factors. They often form multiple macropinosomes when they close [52]. These structures again have a central domain of PIP3 surrounded by a ring of F-actin [19,21].

Thus large, cupped structures — macropinocytic cups, phagocytic cups and circular dorsal ruffles — all have a central PIP3 domain, around which actin polymerizes. This suggests a common mode of formation, as is provided by the stalled wave model. However, many questions of mechanism and function remain.

PIP3 domains are enigmatic [27] (Figure 2). They contain both signalling molecules: active Ras and Rac [53,33], PI3-kinase and PIP3, plus regulators such as RasGAPs [31,54,55] and effectors recruited by PIP3, such as Akt, all associated with an F-actin scaffold. Since Ras and PIP3 are freely diffusible in the membrane, special measures are required to produce high local concentrations in domains. Likely there is a positive feedback loop [47], which may be based on Ras, [33]: for instance, Ras might activate its own GEF, analogous to the activation of SOS by Ras-GTP [56]. Domains may also be sustained by diffusion barriers in the membrane to retain or exclude diffusible molecules. These have been detected in both macrophages and amoebae [57,58]. It is not known how Scar/WAVE is recruited to the edge of domains. One idea is based on a Scar/WAVE recruitment annulus around domains created by overlapping an inhibitory domain of Ras/PIP3 and a slightly larger activating domain of Rac [54].

Even less is known about how the membranes fuse when a macropinosome closes. Fusion in the base of a macropinocytic cup can be explained by stretching and rupture of the membrane, but in the lip it occurs simply when the membranes collide [34]. Similarly, in macrophages macropinosomes can form when membrane ruffles collide [21]. Are membranes of PIP3 domains fusogenic and if so, what is the fusion mechanism?

The evolutionary story of macropinocytosis and its function in normal physiology is still unfolding. The species distribution (amoebozoa and metazoa) suggests an early evolution for feeding [6]. In metazoa this function has largely been displaced by extracellular digestion in the gut, yet macropinocytic absorption in the gut is reported in some animals and mammalian newborns [59]. The potential for macropinocytotic feeding lies latent in many cells [7] and may be called upon in special circumstances, for instance when the supply of amino acids is limiting [15,16]. Further examples from normal cells are to be expected. Macropinocytic feeding in cancer cells can be seen as an inappropriate activation of this ancient feeding mode [17,18]. The role of macropinocytosis in immune surveillance may also be an adaption of its feeding function, in which captured proteins are released from macropinosomes into the cytoplasm for processing and display on immune cells [60]. Less expected is the value of macropinocytosis for immune cells exploring tight spaces, where they can overcome hydraulic resistance literally by drinking their way forward [61]. Finally, there is the proposal dating back to Lewis [3], that macropinocytosis may be used for cleansing the extra-cellular medium, again making use of its conserved digestive function.

In summary, macropinocytosis is an exciting subject with an unusual number of open questions of function and mechanism. Solving these is of intrinsic interest and has promise for medical advance.

## Figures and Tables

**Table 1:** Ras/PI3kinase signalling is required for macropinocytosis

Gene	Fluid uptake compared with control	Comment	References
NF1-	4–20-fold increase	NF1 (neurofibromin) RasGAP deleted in a wild-type strain	[31]
RasG G12T	2–3-fold increase	Oncogenic Ras overexpressed in wild-type strain with intact NF1	[64]
RasG-/RasS-	34%	KO of 2 out of 12 Ras genes	[64]
PI3kinase1-5-	~10%	KO of all 5 Ras-activated PI3K genes	[26,33]
PTEN-	~10%	PTEN reverts PIP3 to PI4,5P2	[33]
Akt-/SGK-	~10%	Proteins called PKB and PKBR1 in *Dictyostelium*	[65]

Fluid uptake as a measure of macropinocytosis was determined using FITC dextran and flow cytometry [29]. The great majority of fluid uptake (~90%) is due to macropinocytosis, with the residue probably due to clathrin-mediated endocytosis. Two different genetic backgrounds were used in these experiments: close to wild-type (DdB) with intact NF1 and low fluid uptake, which is greatly stimulated by deletion of NF1 or expression of oncogenic Ras (top two lines); or a standard laboratory strain (Ax2) with deleted NF1 and high uptake, which depends on Ras, PI3-kinase, PTEN and the Akt/SGK protein kinases (rest of table).

## Perspectives

- The stalled wave model provides a framework for understanding how macropinocytic cups form and close. Ringed recruitment of actin polymerization around PIP3 domains may be a more general organizing principle for cupped structures, including phagocytic cups and circular dorsal ruffles.
- Specific drugs and inhibitors targeting macropinocytosis to the exclusion of other processes are not currently available. New targets for drugs may be revealed by answering key mechanistic questions — how is Scar/WAVE recruited around PIP3 domains; how are domains sustained; how do the opposing membranes fuse when a macropinosome seals?
- Macropinocytosis likely evolved for feeding and lies latent in many mammalian cells. Is it used more widely for feeding by normal cells when amino acids are limiting, for instance in wound healing? Is its high capacity and non-selectiveness exploited by macrophages to cleanse the interstitial fluid of denatured proteins, cell fragments and viruses?
